# Anonymised and aggregated crowd level mobility data from mobile phones suggests that initial compliance with COVID-19 social distancing interventions was high and geographically consistent across the UK

**DOI:** 10.12688/wellcomeopenres.15997.1

**Published:** 2020-07-17

**Authors:** Benjamin Jeffrey, Caroline E. Walters, Kylie E. C. Ainslie, Oliver Eales, Constanze Ciavarella, Sangeeta Bhatia, Sarah Hayes, Marc Baguelin, Adhiratha Boonyasiri, Nicholas F. Brazeau, Gina Cuomo-Dannenburg, Richard G. FitzJohn, Katy Gaythorpe, William Green, Natsuko Imai, Thomas A. Mellan, Swapnil Mishra, Pierre Nouvellet, H. Juliette T. Unwin, Robert Verity, Michaela Vollmer, Charles Whittaker, Neil M. Ferguson, Christl A. Donnelly, Steven Riley

**Affiliations:** 1 MRC Centre for Global Infectious Disease Analysis, Abdul Latif Jameel Institute for Disease and Emergency Analytics (J-IDEA),, Imperial College London, London, UK; 2 NIHR Health Protection Research Unit in Healthcare Associated Infections and Antimicrobial Resistance, Imperial College London, London, UK; 3 School of Life Sciences, University of Sussex, Brighton, UK; 4 Department of Statistics, University of Oxford, Oxford, UK

**Keywords:** Covid-19, SARS-CoV-2, Mobility, Mobile Phone, United Kingdom

## Abstract

**Background:** Since early March 2020, the COVID-19 epidemic across the United Kingdom has led to a range of social distancing policies, which have resulted in reduced mobility across different regions. Crowd level data on mobile phone usage can be used as a proxy for actual population mobility patterns and provide a way of quantifying the impact of social distancing measures on changes in mobility.

**Methods:** Here, we use two mobile phone-based datasets (anonymised and aggregated crowd level data from O2 and from the Facebook app on mobile phones) to assess changes in average mobility, both overall and broken down into high and low population density areas, and changes in the distribution of journey lengths.

**Results:** We show that there was a substantial overall reduction in mobility, with the most rapid decline on the 24th March 2020, the day after the Prime Minister’s announcement of an enforced lockdown. The reduction in mobility was highly synchronized across the UK. Although mobility has remained low since 26th March 2020, we detect a gradual increase since that time. We also show that the two different datasets produce similar trends, albeit with some location-specific differences. We see slightly larger reductions in average mobility in high-density areas than in low-density areas, with greater variation in mobility in the high-density areas: some high-density areas eliminated almost all mobility.

**Conclusions: **These analyses form a baseline from which to observe changes in behaviour in the UK as social distancing is eased and inform policy towards the future control of SARS-CoV-2 in the UK.

## Introduction

Previous studies have highlighted the impact of changes in human mobility on the transmission dynamics of SARS-CoV-2 in China ^1, 2^, Italy ^3^, Brazil ^4^, and elsewhere. To slow the spread of SARS-CoV-2, many countries have imposed social distancing interventions designed to reduce the number of potentially infectious contacts. These interventions also reduce individual mobility. Therefore, monitoring both national and local changes in mobility can provide useful measures of intervention efficacy, especially in the early stages of national epidemics ^5^. 

Human mobility data gathered from mobile phone handsets have been used effectively to measure population response to health crises prior to the emergence of the SARS-CoV-2 ^6, 7^. The high numbers of mobile phone users worldwide make mobile phone data a good proxy for population mobility patterns ^8^. The anonymised and aggregated crowd level location data used from O2 is based on the location of the cell site mast to which devices are near at a point in time ^8^. This is distinct from other companies which are predominantly app based social media and search companies providing location data via the phone operating system and their apps which record the location and estimated accuracy of that location at regular intervals when GPS is enabled on the handset.

The UK government introduced social distancing via a series of specific policies: from 12th March, individuals displaying any COVID-19 symptoms – either a new continuous cough or a high temperature – were asked to isolate at home for 7 days. From 16th March, people were asked to stop all non-essential contact, stop all unnecessary travel, and to work from home where possible. The recommendation of self-isolation for those with symptoms continued, but, in addition, from that time non-symptomatic members of the household were also asked to isolate for 14 days. Schools were closed after 20th March, along with pubs, clubs, restaurants, gyms, and other retail and leisure locations. From 23rd March, government guidance changed further to “you must stay at home”, with people only being allowed to leave their home for essential food shopping, medical necessity, one daily form of exercise, and work if working from home was not possible. The first relaxation of these measures occurred in England on the evening of 10th May 2020 when individuals who could not work from home were encouraged to return to work on 11th May. From 13th May all individuals in England were permitted to engage in multiple trips outdoors for exercise or to visit parks. (See *Extended data*, Table S1 ^9^, which includes links to specific pages on www.gov.uk for each non-pharmaceutical intervention.)

Here we use data from the Facebook application on mobile phones (referred to as Facebook app in the rest of this document) from the COVID Mobility Data Network for the United Kingdom (UK) and Ireland, and mobile phone data from O2, which is not only anonymised and aggregated to crowd level data but which is also further extrapolated to give a picture of the full UK population, to describe recent changes in mobility across the UK and potential implications for SARS-CoV-2 transmission.

## Methods

### Data sources

We received data via the Facebook Data for Good program ^10^ describing the daily number of trips starting within a tile, and the sum of the length of all trips starting within a tile, where a tile is defined by a grid that Facebook applies to the Earth’s surface in order to aggregate the movement of its users ^11^. Each tile is approximately 25 km ^2^. A trip in the Facebook data is defined as the movement of a handset from one tile to another between two subsequent updates of the geolocation data (the time period between updates is defined by mobile phone operating systems). The latitude and longitude of the centroid of each tile was provided and these were used to aggregate data from the tile level to the local authority district (LAD) level for consistency with the mobile network data (described below). The data were used for 10th March - 22nd May 2020 inclusive for Facebook and for 1st February - 5th May 2020 inclusive for the mobile network.

Facebook also provided data on the daily number of active users of their app, with GPS location services enabled, within different locations. We also aggregated these data to UK LADs and calculated the Facebook population size in each LAD relative to the mean in the first week of data (10th - 16th March 2020 inclusive). To adjust for changing Facebook population size over time ( *Extended data*, Figure S2 ^9^) the number of daily trips per LAD and the daily total distance travelled per LAD were divided by the Facebook population size per LAD relative to the first week of data.

In addition to the Facebook data, we received anonymised and aggregated crowd level mast data from O2 (which is further modelled to the UK population) detailing the number of trips starting within each UK local authority district for 1st February - 5th May 2020 inclusive. A trip is created when a device moves from one overlapping group of cells to another (where a cell is an area of coverage provided by a single mobile network antenna) and remains with the group of cells for long enough to indicate the device is stationary. As these are mast data, all mobile phones registered to the data provider’s network are recorded, not just smartphones with GPS tracking enabled.

### Data comparison and normalisation

To enable comparison of the mobile network data with the Facebook data, we defined a baseline level of mobility as the mean daily number of trips within the week 10th - 16th March 2020 per LAD. We then calculated the percentage change in mobility for the Facebook and mobile network data compared to this baseline.

We inspected the data on mean daily number of trips within the following spatial regions: individual countries of the UK, specific cities (defined by the boundaries of their constituent LADS) which were selected because they are the largest city in each of the four countries of the UK respectively (London, Cardiff, Glasgow, and Belfast), LADs, and finally we grouped the LADs into quartiles of population density (throughout, the quartile of lowest population density is referred to as 1, whilst quartile 4 is the quartile of greatest population density). The location of population density quartiles is shown in *Extended data*, Figure S1 ^9^. Data describing the population per LAD were provided by LandScan ^12^.

### Model fitting

We fitted a segmented-linear model ^13^ to the percentage relative to baseline for the nationally aggregated Facebook data against time. The segmented-linear model divides the data into multiple time components split by “breakpoints”, then, using linear regression fits a straight line to each separate component. The dates of each breakpoint are included as parameters of the model. The minimum size of a component allowed was chosen to be three data points to minimise overfitting.

Due to the categorical difference between levels of mobility on weekdays, Saturdays, and Sundays, we only fitted the model to weekdays. Bank holidays were also omitted from the data during the fitting as they were markedly different to the other weekdays but were not considered to be representative of any longer-term trends in the data.

We investigated segmented-linear models with 0, 1, 2, 3, 4, 5 and 6 breakpoints. For each model, all possible permutations of breakpoints were considered subject to the constraint of there being a minimum of three data points per component. For each permutation the other parameters of the model were fitted assuming log-normal errors for the number of trips as a percentage compared to baseline. The relative weighting of points was estimated using inverse-variance weighting, where relative variance was estimated as the daily number of trips. The permutation with the highest likelihood was then considered the best fit for the model. Comparisons of the segmented-linear models (with different numbers of breakpoints) were made using the corrected Akaike information criterion (AICc), with the most parsimonious model being the one with the lowest AICc.

Finally, we also investigated differences in the distribution of distance travelled per journey in areas of high and low population density, and how these distributions changed following the introduction of social distancing measures. The shape of these distributions was defined by smoothing over the data points using a Gaussian kernel.

All the analysis was conducted using the R statistical programming language ^14^; the code used to plot the figures and run the analyses is publicly available at https://github.com/mrc-ide/covid-uk-mobility-report and has been archived with Zenodo ^9^.

## Results

### Changes in mobility over time

**Figure 1.**
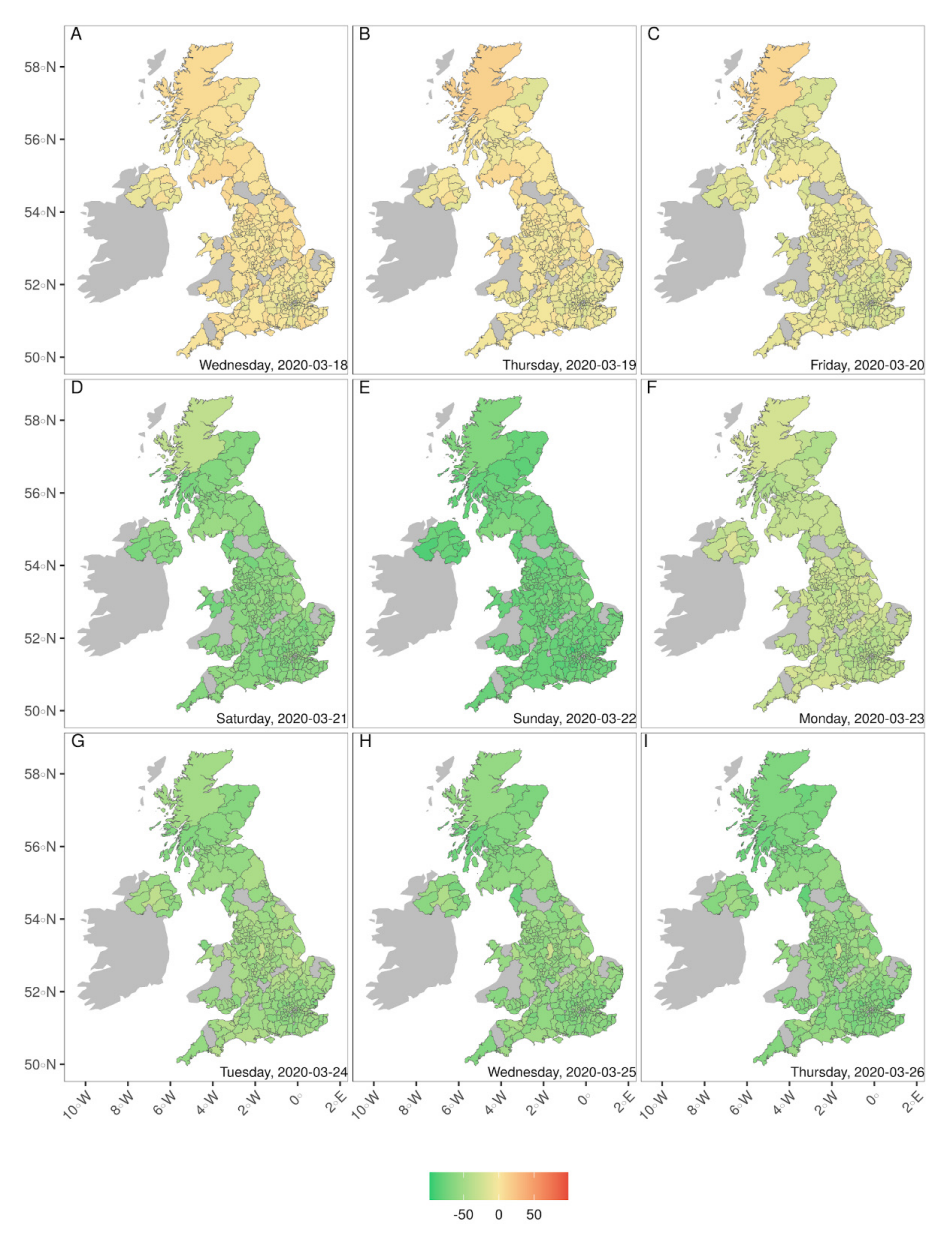
Rapid reduction in mobility in the UK from the 18th March 2020 ( **A**) through to the 26th March ( **I**). Colour shows percentage change in daily number of journeys compared to the mean in the week 10th-16th March 2020 inclusive, by origin tiles that consistently reported data each day. Sufficient data were not available for tiles in the grey area. Note that (C) and (D) are weekend days and there was an increase in overall mobility on the 23rd March (see also Figure 2).

**Figure 2.**
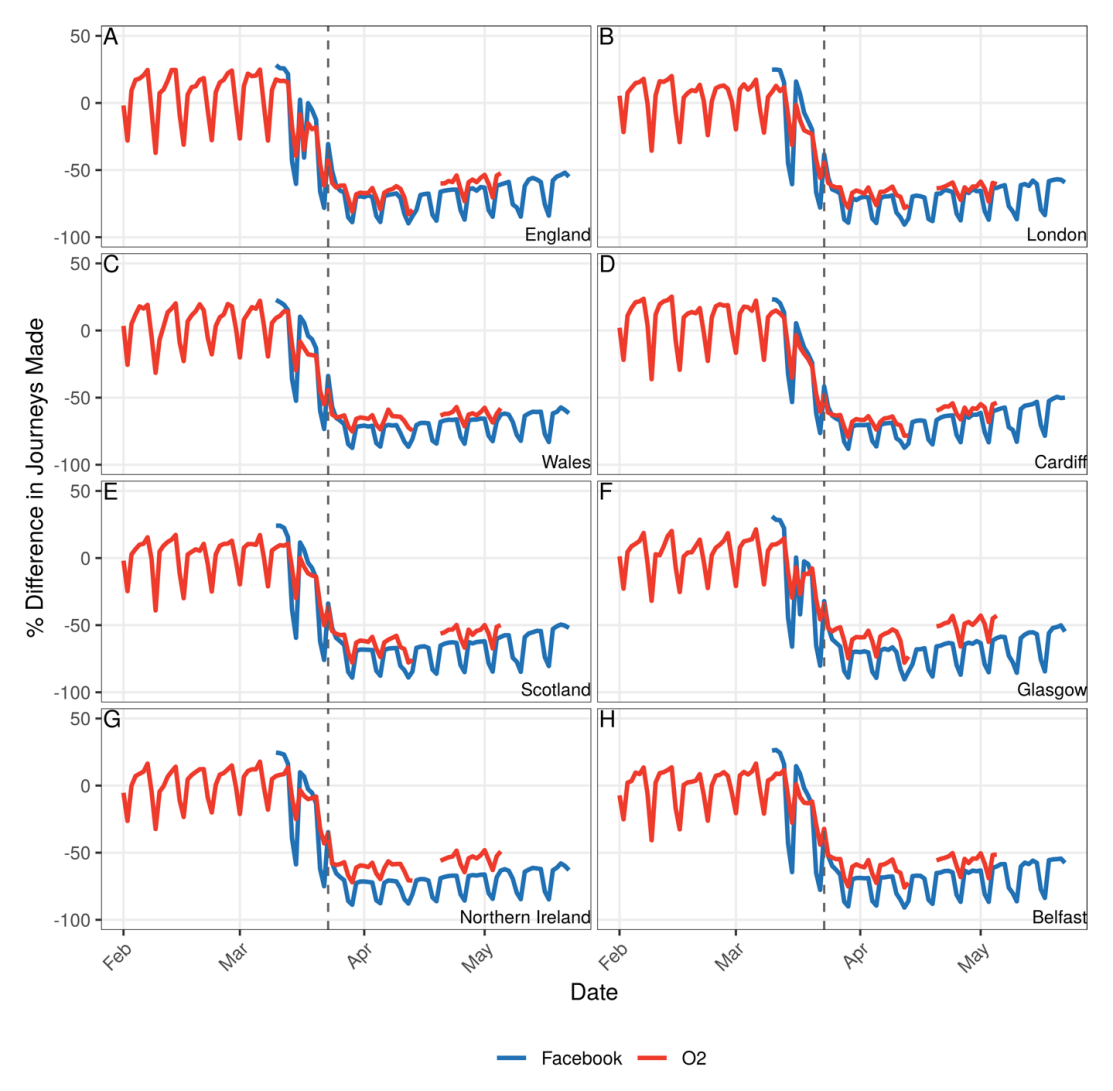
Consistent changes in mobility observed between Facebook data and mobile phone data. Change in movement over time as a percentage of baseline movement for the four home countries within the UK and their largest city for Facebook data (blue) and mobile phone data (red). Baseline movement defined as the mean number of journeys starting within a small unit within each city from 10th-16th March 2020 inclusive. The dashed vertical line at 23rd March indicates when the most stringent lockdown measures were imposed.

**Figure 3.**
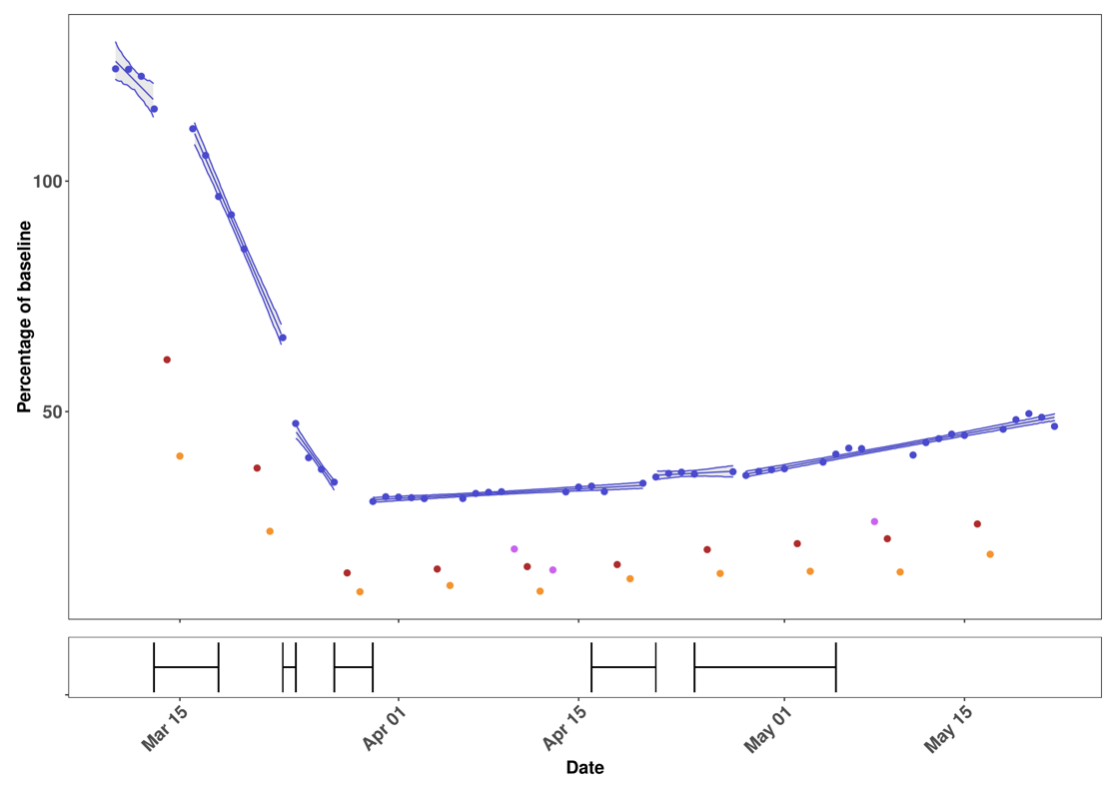
Fit of the segmented-linear model with 5 breakpoints to the percentage relative to baseline of number of trips against time (top panel). Weekdays (blue), Saturdays (red), Sundays (orange) and bank holidays (purple). Bottom panel shows univariate 95% confidence regions for each breakpoint.

The mean number of trips per local authority district (LAD) made in the UK by users of the Facebook mobile phone app dropped substantially between the 18 ^th^ and 26th of March inclusive ( Figure 1), from around 99% of the baseline to around 37%. The number of trips was still close to the baseline on Friday 20th March (the last day in which schools were open, other than to vulnerable children and the children of key workers, see *Extended data*, Table S1 ^9^) at approximately 87% of the baseline, then dropped substantially over the weekend of 21st - 22nd March to about 30% of the baseline number of daily trips per LAD. This was the first weekend during which all pubs, clubs, bars, gyms, etc. were closed ( *Extended data*, Table S1 ^9^). We see a slight increase in the mean number of trips per LAD on Monday 23rd to 67% of the baseline. On the evening of Monday 23rd of March the government messaging changed from “we advise you to stay home” to “you must stay home”. From Tuesday 24th to Thursday 26th of March we then see a greater decrease in the number of daily journeys made per LAD to approximately 40% of the baseline across the UK on working days, and approximately 15% of the baseline on the following weekend. As a percentage of the number of daily trips made per LAD, this difference between weekdays and weekends remains approximately constant from the 26th of March until the last day of data we considered: the 22nd of May ( Figure 2 and Figure 3).

### Mobility variation by location and data source

There was little variation in mobility reductions either by data source, city (London, Cardiff, Glasgow, Belfast), or country of the UK ( Figure 2). There were clear weekend effects in all areas (see the previous paragraph) and small variations from day to day ( Figure 2); however, trends across different locations were highly consistent. For example, on the Wednesday of the first full week post-lockdown (the 31st of March 2020) the city with the greatest number of daily trips relative to the baseline was Belfast according to the Facebook data and Glasgow according to the mobile phone data (30% and 35% of the baseline respectively), and the city with the fewest number of daily trips relative to the baseline on the same day were Glasgow according to the Facebook data and Belfast according to the mobile phone data (29% and 33% of the baseline, respectively). On average, the Facebook data suggested fewer trips were made following the social distancing interventions than the mobile phone data.

**Figure 4.**
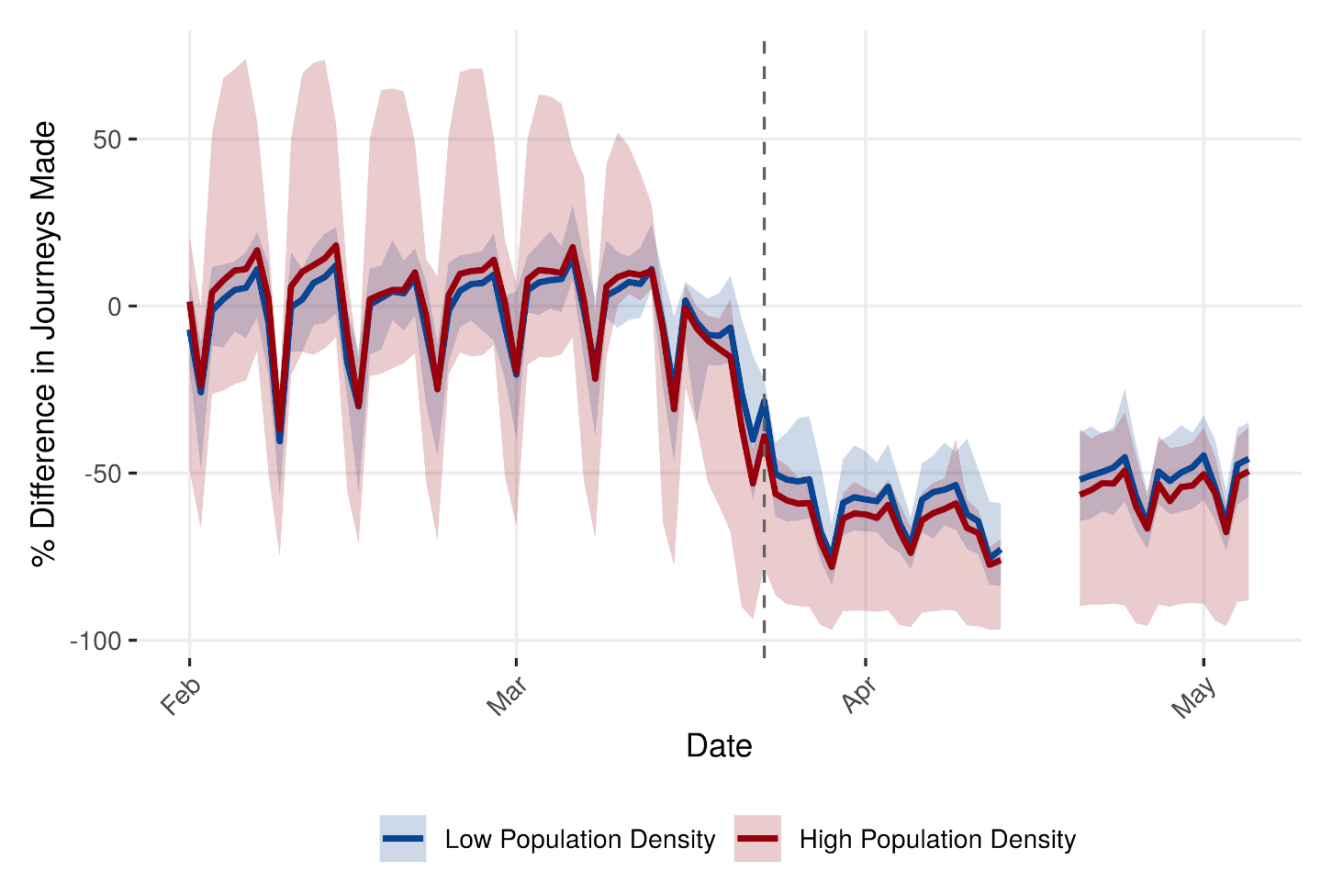
We ranked each UK local authority district by population density and determined the corresponding quartile for each local authority district, with lower population density in quartile 1 and higher population density in quartile 4. The shaded region is the range of percentage differences in journeys made at each time point for both lower quantile (low population density) and upper quantile (high population density) using the mobile data. Solid lines are the median difference from baseline within each quartile. The dashed line on 23rd March is when the most stringent lockdown measures were imposed.

Following the initial reduction in mobility coinciding with the introduction of social distancing measures, there has been a gradual increase in the number of daily trips made by Facebook and mobile phone users, starting on approximately the 1st of April and continuing until the 22nd of May ( Figure 2– Figure 4). Using the Facebook data, we fitted multiple segmented-linear models (see *Methods*) to the number of daily journeys made per LAD as a percentage relative to the baseline (mean number of daily trips per tile in the week 10th – 16th of March). A segmented-linear model with five breakpoints was the most parsimonious ( Figure 3) with breakpoints on 13th March, 23rd March, 27th March, 20th April, and 27th April. However, a four-breakpoint model had only a marginally higher AICc (-300.68 vs -299.86) and so could not be rejected ( *Extended data*, Table S2 ^9^). For the period between the 27th March and 20th April there has been a statistically significant increase in the number of daily trips (p-value < 0.001), with mobility (as a percentage relative to the baseline) increasing at a rate of 0.15 percentage points per day. For the period since the 27th April this rate of increase has been 0.51 percentage points per day. We conclude that since the beginning of April there has been a gradual increase in the number of daily trips made per LAD and that this rate has increased recently.

There was a greater decrease in the median number of trips made within high-density populations compared with low-density populations ( Figure 4), from 91% to 47% of the baseline in low-density populations, and 90% to 40% of the baseline in high-density populations between the 18th and the 26th of March. Prior to the beginning of lockdown on the 24th of March, there was a higher median mobility, more pronounced weekend effects (greater decrease in mobility at the weekend than on weekdays), and a greater range in mobility within high-density populations compared with populations living in regions of lower population density. However, after the lockdown the reduction in mobility in high- and low-density populations was comparable on weekends, and slightly higher on weekdays in low-density populations (e.g. 43% and 38% of the baseline in low- and high-density populations respectively on the 31st of March 2020). Also, the range of reductions in mobility in high-density populations was not symmetric about the median: some high-density populations reduced their levels of mobility by almost 100% compared to baseline levels, whereas the range in low-density population was narrower and approximately symmetric about the median pre and post-lockdown.

**Figure 5.**
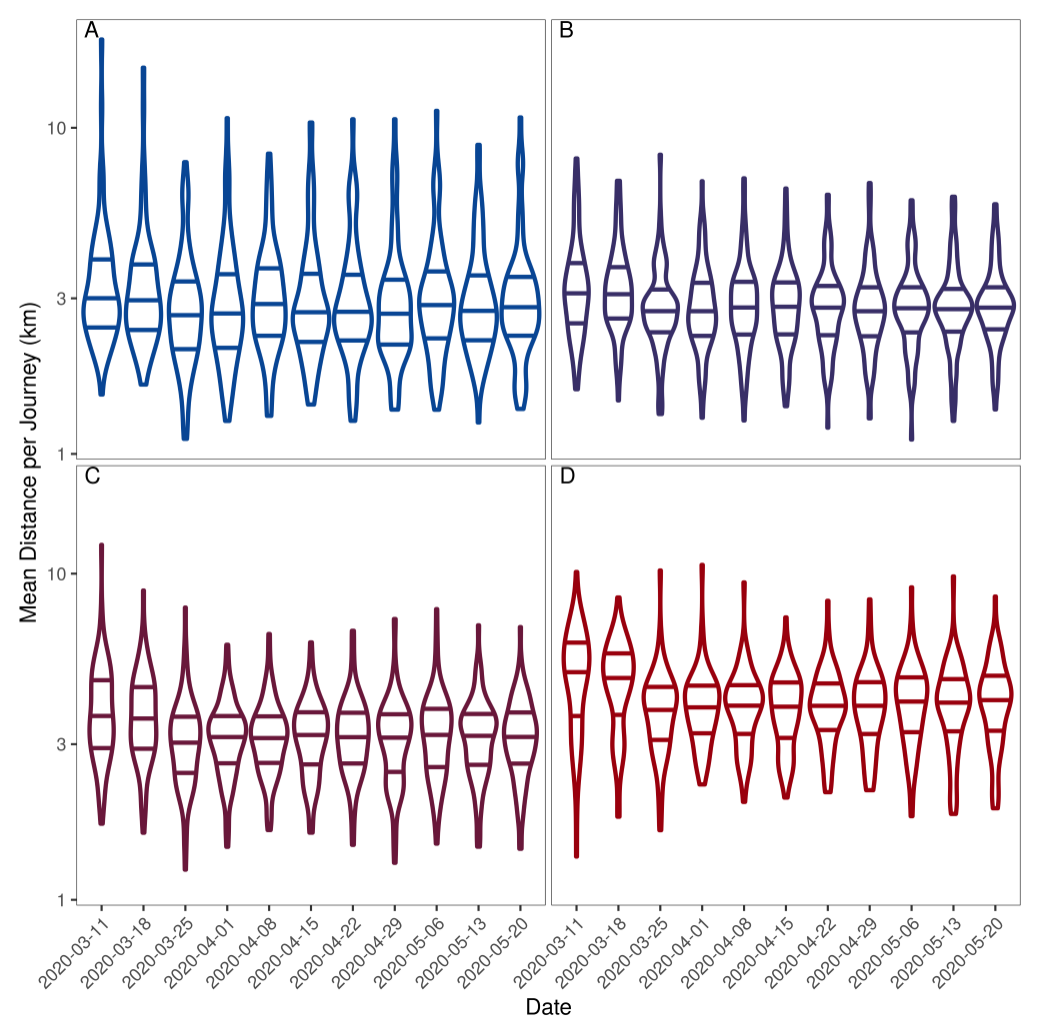
Distribution of mean journey lengths per LAD on each Wednesday in the Facebook data for areas of increasing population density. ( **A**– **D**) Journey length distributions for each quartile of population density from lowest ( **A**) to highest ( **D**). The y axis is on a log10 scale and the median and interquartile range for each distribution is shown by the horizontal lines. A Gaussian kernel is used to define the shape of the distributions which are truncated at the minimum and maximum point in the data.

We observed similar changes in the distribution of journey distances ( Figure 5) to those described above for the number of journeys. In the two lowest quartiles of population density ( Figure 5A, B), the decrease after lockdown was less and the median journey distance was lower at all time points than in the most densely populated LADs. However, the range of journey distance was greater both before and after the lockdown in the lowest density populations ( Figure 5A) than in high-density populations ( Figure 5D).

## Discussion

The sequence of policy decisions implemented up to and including the evening of the 23rd of March 2020 was successful in reducing mobility in the UK, measured as the number of journeys made each day, which may be a good proxy for non-household social interactions. According to both the Facebook and mobile network data there does not appear to have been substantial variation in regional adherence to these interventions, nor was there a noticeable difference between adherence in low and high population density areas. It appears that mobility began to decrease around one week before the lockdown was enforced in the UK on the evening of 23rd of March, but that the sharpest drop was after that date. A gradual increase in the number of trips made each day started at the beginning of April and has continued since that time. This rate of increase may have accelerated somewhat, as indicated by our segmented linear model fits, but the currently estimated rate is that the population is recovering only ~0.5% of prior mobility levels per day.

Interestingly, the degree of synchrony observed in the UK response to lockdown described here has not always been observed elsewhere. For example, state-level mobility in the USA shows much greater variability ^15^ as does between-borough movement in New York City ^16^, whereas both Italy ^3^ and Brazil ^4^ have observed considerably more spatially consistent mobility trends during the course of the SARS-CoV-2 epidemic, similar to what we report here for the UK. This variation in mobility may have resulted in differences in the transmission of SARS-CoV-2 and therefore could explain some of the intra and international variation in cases and deaths related to SARS-CoV-2 infection ^3, 4, 15, 16^.

However, there are slight differences in the median journey distance between high and low population density areas before and after the 23rd of March. This may be because people living in small towns and villages have less far to travel to reach their workplace and essential shops than people who live or work in cities. The greater decrease in median journey distance within densely populated areas during the lockdown possibly reflects the reduced number of people commuting in and out of the centre of major cities.

This study has some limitations. Firstly, data were only reported for locations with sufficiently high numbers of reporting handsets to ensure journeys were not identifiable. This resulted in a subset (approximately 90%) of UK LADs being included in our analyses. Secondly, it is difficult to assess how representative our data are of the wider population not using the Facebook app or O2 as a mobile phone provider. However, it is reassuring that the trends in mobility change are largely consistent between the two data sets. Thirdly, the data sets record handset movement in terms of the number of journeys made and the average distance travelled, which, if being considered as a proxy for transmission, may not always capture the true changes in social contact behaviour. However, we note that decreases in mobility were closely followed by a plateauing and then reduction in the daily case and death rate in the UK ( https://coronavirus.data.gov.uk/). Finally, there is no obvious functional form with which to fit the pattern of recovery of mobility following the lockdown. Therefore, we used a segmented linear regression approach to identify potential breakpoints. During periods of gradual change, these breakpoints may not be reflective of actual step-changes in population behaviour.

We note that Facebook data, which uses GPS location, is collected at a finer spatial resolution compared with the mobile network data, which is at mast location level. However, mobile network data include all types of mobile phones registered to the mobile network, not just smart phones with the Facebook app installed and geolocation services enabled, and is therefore likely to include a larger, and more representative, sample of the population. These differences may account for the difference in change in mobility between the two data sets seen in Figure 2. Comparison with other mobility data sets such as Google handset location data ( https://www.google.com/covid19/mobility/) and Apple route finding data ( https://www.apple.com/covid19/mobility) was beyond the scope of this project because neither of these data sets had sufficient coverage at the subnational level.

Here we have demonstrated the utility of mobile phone data for monitoring changes in human mobility, by assessing how it has changed in response to the COVID-19 epidemic in the UK. We see that whilst adherence to movement restrictions was initially high and geographically consistent, it is waning over time. It will be important to continue to monitor changes in mobility as the epidemic progresses, to inform policy towards limiting the spread of SARS-CoV-2.

## Data availability

### Underlying data

The raw data needed to replicate these analyses has not been made public by the data providers, meaning we are forbidden from sharing it with this paper. However, the reader can apply for access to the Facebook data through the Geoinsights platform here: https://dataforgood.fb.com/, or by emailing disastermaps@fb.com or diseaseprevmaps@fb.com. Details on how to access these data are available at https://dataforgood.fb.com/tools/population-density-maps/. 

Mobile phone operator data was provided by O2 Motion. More details on how to apply to access these data can be found at https://www.o2.co.uk/business/solutions/data-mobile/o2-motion.

### Extended data

Zenodo: mrc-ide/covid-uk-mobility-report: Initial Release. https://doi.org/10.5281/zenodo.3865406 ^9^. ****

This project contains the following extended data:

- wellcome_open_mobility_supp_mat.pdf (supplementary material containing Tables S1 and S2, and Figures S1 and S2).
- Code used to perform the statistical analyses and produce Figure 1– Figure 5, S1, and S2.

Data are available under the terms of the Creative Commons Zero "No rights reserved" data waiver (CC0 1.0 Public domain dedication).
