# Supplementary material for: Anonymised and aggregated crowd level mobility data from mobile phones suggests that initial compliance with COVID-19 social distancing interventions was high and geographically consistent across the UK
Source: Wellcome Open Res. 2020 Jul 17;5:170. [Version 1] doi: 10.12688/wellcomeopenres.15997.1 (PMC7479499; doi:10.12688/wellcomeopenres.15997.1)
Supplement: Peer review history

# Peer review: Reviewer response for version 1

**DOI:** 10.21956/wellcomeopenres.17546.r40111

**Published:** 2020-09-08

**Authors:** Andrew Schroeder

**Affiliations:** 1 Direct Relief, Santa Barbara, CA, USA; 2 WeRobotics, Ann Arbor, MI, USA

The authors have demonstrated clearly and convincingly based upon two distinct sources of digital mobility data that populations throughout the UK responded to Covid-19 related social distancing orders by dramatically reducing rates of physical mobility relative to a baseline mobility level of early March, 2020. One data source is derived from the O2 mobile phone provider and the other is derived from aggregated users of the Facebook smart phone app with location services enabled on an opt-in basis. 

 The two data sources, O2 and Facebook, demonstrate remarkable consistency between them, and between geographic regions throughout the UK. The consistency of agreement between data sources, in terms of rates of mobility change relative to baseline and the timing of key changes in the rate of mobility, is highly significant with only very minor regional variations. The importance of this finding pertains to the issue of general representativeness of the data, one of the key issues in the use of digital mobility data for physical distancing measurement. Comparative research into digital mobility data, particularly in terms relative representativeness of the population sample, is still in early stages, with considerable variance globally. (See Kishore N, Kiang M, et al,: Measuring mobility to monitor travel and physical distancing interventions: a common framework for mobile phone data analysis. The Lancet Digital Health; Sept 01, 2020 ^1^) The contribution of this article to the advancement of comparative digital mobility research is therefore substantial.

 By showing that O2 and Facebook closely correlate on timing and magnitude of mobility changes the authors increase confidence in the digital mobility signal substantially, even though the causes of the variations they did detect are not explained sufficiently. Nevertheless, the regional correlation overall is strong enough to merit the author's conclusion that the UK responded to physical distancing orders with a high degree of unanimity throughout the country. 

 The one area which may require further inquiry relates to the causes of timing in mobility change. Whereas the authors note highly specific responses to the wording of distancing orders in March, with variances measured in days, there is no similar attention paid to the clear erosion in rates of mobility reduction which occurred in April. This is curious given that the erosion is not only evident in both the O2 and the Facebook data, but also potentially significant in terms of epidemiological factors noted by the authors, including the viral contact rate. Likewise, the change in April occurred well before formal policy changes were enacted on 11 May, and bear further scrutiny to understand what may be important distinctions between short-term health policy responses and longer-term behavioral shifts.

Is the work clearly and accurately presented and does it cite the current literature?

Yes

If applicable, is the statistical analysis and its interpretation appropriate?

Yes

Are all the source data underlying the results available to ensure full reproducibility?

Yes

Is the study design appropriate and is the work technically sound?

Yes

Are the conclusions drawn adequately supported by the results?

Yes

Are sufficient details of methods and analysis provided to allow replication by others?

Yes

Reviewer Expertise:

My area of expertise is focused on spatial analysis and applied digital informatics for humanitarian aid and public health.

I confirm that I have read this submission and believe that I have an appropriate level of expertise to confirm that it is of an acceptable scientific standard.

## References

[1] : Measuring mobility to monitor travel and physical distancing interventions: a common framework for mobile phone data analysis. *The Lancet Digital Health*.2020; 10.1016/S2589-7500(20)30193-X 10.1016/S2589-7500(20)30193-X PMC746256532905027

# Peer review: Reviewer response for version 1

**DOI:** 10.21956/wellcomeopenres.17546.r39607

**Published:** 2020-08-17

**Authors:** Rowland Kao

**Affiliations:** 1 Roslin Institute, University of Edinburgh, Edinburgh, UK

This is a useful paper, outlining changes in mobility across geographical regions of the UK. It also analyses these data with a simple model which can be used to break up changes in mobility patterns in a sensible and parsimonious way. 

 I only have a few comments to make about the paper. 

1. The authors at one point mention Ireland but really only seem to analyse Northern Ireland, which is in the UK.
2. The paper combines data from O2 and Facebook - these of course cover different aspects of the data, and it is very good to know that despite this, there is quite good consistency in their temporal signatures. However there do appear to be some differences - it might be useful to point out how they are different (e.g. the Facebook data seem to show greater variation across days of the week and between the pre and post lockdown periods - is this correct, and if so, is there a known reason why
3. It is mentioned in the methods that the area of tiles is about 25km ^2^. If I remember correctly the tiles are determined by lat and long rather than a fixed distance and so will vary over the globe? if this is correct, it is probably worth stating. 
4. I may have misunderstood the method but why are there gaps in the piecewise linear model as shown in figure 3?
5. A fair bit of the discussion hinges on the assumption that the mobility data 'may be a good proxy for non-household interactions.' and there there was no noticeable difference to adherence. I would challenge this as there is no real evidence that this is true. The restriction one what are fairly long distance movements is one thing. But adherence is a different thing altogether. I would argue that we really just don't know.
6. Related to this, the authors note that "we note that decreases in mobility were closely followed by a plateauing and then reduction in the daily case and death rate in the UK". This is true, but the mobility measures at the time were accompanied by social distancing measures. Given that these occurred at about the same time, its hard to say just how much mobility reduction had to do with this, as opposed to say the 2m rule, which was independent of it. A further suggestion that mobility may not have had a big effect on transmission directly, is that even though mobility as shown here was increasing steadily over the lockdown period, there does not seem to have been an equivalent increase in R. Of course other factors likely come into play there as well, but I would be cautious in linking the two. Of course, reduction in mobility has a big effect on geographical spread (or should) and this should probably be mentioned in the paper.
7. About the differences in median journeys between high and low density areas - I wonder if this may be partly due to different proportions of essential workers in the different areas - i.e. more people might still have to travel to work despite lockdown, rather than this simply being because of differences in commuting distance. You could see if this is the case by looking at the distribution of distance changes per journey. i.e. if a greater proportion of journeys stayed consistent for example, this would suggest some people maintaining similar work patterns.  

Is the work clearly and accurately presented and does it cite the current literature?

Yes

If applicable, is the statistical analysis and its interpretation appropriate?

Yes

Are all the source data underlying the results available to ensure full reproducibility?

Yes

Is the study design appropriate and is the work technically sound?

Yes

Are the conclusions drawn adequately supported by the results?

Partly

Are sufficient details of methods and analysis provided to allow replication by others?

Yes

Reviewer Expertise:

Infectious disease epidemiology, including impact of mobility patterns

I confirm that I have read this submission and believe that I have an appropriate level of expertise to confirm that it is of an acceptable scientific standard.
